# Audacious Hitchhikers: The Role of Travel and the International Food Trade in the Global Dissemination of Mobile Colistin-Resistance (*mcr*) Genes

**DOI:** 10.3390/antibiotics9070370

**Published:** 2020-07-01

**Authors:** Jouman Hassan, Issmat I. Kassem

**Affiliations:** 1 Department of Nutrition and Food Sciences, Faculty of Agricultural and Food Sciences, American University of Beirut (AUB), Riad El Solh, Beirut 1107 2020, Lebanon; jwh03@mail.aub.edu; 2 Center for Food Safety and Department of Food Science and Technology, University of Georgia, 1109 Experiment Street, Griffin, GA 30223-1797, USA

**Keywords:** *mcr*, colistin, antibiotic resistance, antibiotic-stewardship, travel, trade, food, global dissemination

## Abstract

Colistin, a last-resort antibiotic, has been used in controlling infections caused by multidrug-resistant Gram-negative bacterial pathogens. However, recent reports showed a global dissemination of mobile colistin-resistance *(mcr*) genes, genetic elements that encode resistance to colistin, which has raised public health concerns. These *mcr* genes threaten the effectiveness of colistin and could limit therapy options for complicated infections. Despite global attention, many facets of the molecular epidemiology of *mcr* remain poorly characterized. Here, we focus on the role of travel and the international food trade in the dissemination of *mcr* to countries where these genetic elements and/or colistin resistance are relatively limited in prevalence. We present evidence from the literature on the acquisition of *mcr* during travel, and the carriage of these genes back to travelers’ countries. We also highlight the potential transmission of *mcr* via imported foods. These observations emphasize the magnitude of efforts that are needed to control the spread of *mcr,* and further highlight the challenge of antimicrobial resistance and the urgent need for coordinated global action.

## 1. Colistin Resistance and *mcr* in Brief

Antimicrobial resistance (AMR) poses a serious threat to public health across the globe. Complicated infections attributed to multidrug-resistant (MDR) and extensively drug-resistant (XDR) bacterial pathogens are predicted to increase in frequency. As these pathogens rapidly evolve to resist currently available antibiotics, treatment options are becoming more limited. This problem is further compounded by weak incentives for investments in- and discovery of novel antibiotics [1]. Therefore, it is imperative to globally enhance antimicrobial stewardship in order to maintain the efficacy of available antibiotics while seeking alternative therapies. This is logically more urgent in the case of antibiotics that have been dubbed “last-resort”, a label that highlights their vital importance in combating critical infections. Colistin (polymyxin E) is a last-resort antibiotic, and it is one of the limited options available for treating carbapenem-resistant *Enterobacteriaceae* infections. It could be considered very revealing that colistin was reintroduced to human medicine to fight these infections despite its established toxicity [2,3]. However, the effectiveness of colistin is threatened by the recent emergence and global dissemination of mobile colistin-resistance (*mcr*) genes, genetic elements that encode resistance to this antibiotic [4,5]. The first plasmid-mediated colistin-resistance gene, *mcr-1*, was reported in 2016, when it was detected in *Escherichia coli* isolated from a pig in China [4]. Notably, it was shown that *mcr-1* was transmissible between bacterial strains, and the authors predicted that *mcr-1* would emulate other resistance genes that spread globally. Indeed, *mcr-1* has now been detected on at least five continents, and it was shown to occur in a variety of genomic backgrounds, and a wide range of niches and hosts [5,6]. Furthermore, nine different homologues (*mcr-1* to -*9*) and multiple variants of some of the genes (e.g., *mcr-1.2* to *-1.8*) were described to date. Despite global interest and potentially severe repercussions on antimicrobial stewardship, certain aspects of the molecular epidemiology of *mcr* remain poorly characterized [7,8,9,10]. These include its origins, potential reservoirs, bacterial-fitness costs, vehicles of transmission, and other factors that facilitate the dissemination of these genes. This article highlights the role of travel and the food trade in the spread of *mcr* to different countries, including those with relatively good antimicrobial-stewardship programs, and/or low colistin use and resistance. Specifically, it discusses evidence from the literature on the travel- and food-associated dissemination of *mcr* to Europe, the USA, Canada, and Japan. The genes appear to be audacious hitchhikers of human travelers and foods, which constitute important routes of transmission that should not be overlooked in the epidemiology of colistin-resistant infections. Given that travel and the food trade bridge different countries, we emphasize that controlling *mcr* dissemination could be difficult in the absence of a coordinated global effort.

## 2. Dissemination of *mcr* to European Countries via Travelers

Several studies linked international travel to *mcr* acquisition by Europeans who transported the genes back to their home countries. For example, a prospective study was conducted in the Netherlands to assess the possible acquisition of extended-spectrum-β-lactamase (ESBL)-producing *Enterobacteriaceae* by travelers. Fecal-swab samples were collected immediately before travel and within one to two weeks of travelers’ return; *mcr-1* was detected in six ESBL-producing *E. coli* isolated from six different travelers [11]. The travel destinations of individuals who carried *mcr-1* back to the Netherlands were Peru, Bolivia, Colombia, China, Tunisia, Thailand, Vietnam, Laos, and Cambodia. The travelers were between 25 and 62 years old, and the duration of their travels ranged from 8 to 40 days. Notably, five individuals reported contracting travelers’ diarrhea while abroad. In another study on Dutch travelers, DNA in the fecal samples from 122 healthy individuals was screened before and after long-distance travel; *mcr-1* was detected in six travelers who had acquired the gene after visiting countries in Asia and Africa, namely, Thailand, Vietnam, Indonesia, Tanzania, and India. The travelers were between 24 and 69 years old, and the duration of their travels ranged from 5 to 35 days. The authors concluded that there was an increase in the prevalence of *mcr-1* in Dutch citizens after travel that could have contributed to the dissemination of the gene in the Netherlands [12]. Similar but less-controlled observations were reported in Sweden, Switzerland, and Norway. Specifically, two *mcr-1*-positive *E. coli* were isolated from fecal samples collected from Swedes who had traveled to Asia [13]. Furthermore, *mcr-1*-positive *E. coli* (sequence type ST10) was isolated from the fecal matter of a Swiss traveler returning from India [14]. In Norway, *mcr-1*-positive *E. coli* was isolated from a traveler who had visited India and was suffering from travelers’ diarrhea [15].

Transmission to European countries may not have been restricted to *mcr-1* in *E. coli*. In a retrospective study from the United Kingdom, *mcr-1*-positive *E. coli* and *Salmonella* spp. were recovered from individuals that had a history of travel to countries in Asia and North Africa, namely, Borneo, Cambodia, Egypt, Hong Kong, Malaysia, Singapore, Thailand, and the United Arab Emirates [16]. Similarly, an *mcr-1*-positive *Salmonella* Typhimurium and other *Salmonella* isolates that harbored *mcr-3* were detected in Denmark among patients with a history of travel to Thailand and Vietnam [17,18]. Furthermore, in Denmark, *mcr-3*-positive ESBL-producing *E. coli* (sequence type ST131) was isolated from a bloodstream infection in a patient who had traveled to Thailand two months prior to hospital admission [19]. Notably, *mcr-1*-positive *Enterobacter cloacae* was detected in an Algerian patient hospitalized in France. Given that *mcr-1*-positive *E. cloacae* had been previously described only in Asia at the time, it was speculated that the bacterium could have been acquired outside of France [20]. The spread of *mcr* by travelers is not restricted to Europe, and there is evidence that shows similar transmission to other countries.

## 3. Dissemination of *mcr* to USA, Canada, and Japan via Travelers

In Connecticut, USA, *mcr-1* was detected in non-Shiga-toxin-producing *E. coli* O157, isolated from fecal samples taken from a pediatric patient. The patient had visited the Caribbean for almost two weeks and exhibited fever and bloody diarrhea two days before returning to the US mainland. The authors suggested that *mcr-1* could have been acquired during travel, partially because the *mcr-1*-positive isolate was only the fourth detected in patients in the US mainland [21]. Additionally, *mcr-1*-positive *E. coli* was isolated from urine samples collected from four patients (two males and two females) in Michigan, USA. Patients’ age ranged from 19 to 77 years, and they had previously traveled to Kenya, China, Lebanon, Mexico, and Western Europe within six months prior to the detection of *mcr-1* in their samples [22]. Moreover, multidrug-resistant *Salmonella enterica* (sequence type ST34) was identified as a carrier of *mcr-3.1*. The isolate dated back to 2014 (prior to the first report of *mcr-1* in China) and was isolated from the feces of an 18-year-old male patient who had visited China two weeks prior to experiencing diarrhea [23].

Similarly, *mcr-1*-positive *E. coli* (sequence type ST3944) was isolated from the urine of a Canadian who had travelled to China for two weeks. The traveler, a 61-year-old male, was hospitalized for acute urinary retention, and received catheterization in China. The traveler suffered from complications after the catheter was removed, and he required intravenous antimicrobial interventions, but his symptoms persisted upon his return to Canada. The authors suggested that *mcr-1* could have been acquired in China because the gene was also reported, albeit at a low frequency (1%), in patients at the Chinese hospitals that had provided care to the traveler [24].

Notably, in a controlled study, 19 individuals were screened before and after travel from Japan to Vietnam; *mcr-1* was detected in *E. coli* carried by three travelers, and the authors concluded that short-term travel (4–12 days) was sufficient for the acquisition and transport of *mcr-1* by travelers returning to Japan [25]. Taken together, multiple lines of evidence indicate that *mcr* genes are transported by humans who were exposed abroad to *mcr*-carrying bacteria in a variety of matrices, including hospitals, recreational waters, animals, farms, and contaminated food [6,7,8,26,27]. The last of these may also serve as a vehicle of *mcr* transmission between countries in a traveler-independent manner, namely, via the food trade.

## 4. Dissemination of *mcr* via the International Food Trade

*Enterobacteriaceae* that carry *mcr* genes can colonize food animals and contaminate animal carcasses and associated meat products. For example, eight isolates (19.5%) from chicken in Brazilian markets tested positive for the presence of *mcr-1*, and the gene was also detected in three *E. coli* isolates from retail chicken meat in the Netherlands [28,29]. In Vietnam, *mcr-1* and *mcr-3* were detected in *E. coli* isolated from meat (pork and chicken) and seafood (fish and shrimp) products [30]. Therefore, contaminated food, animals, and meat can serve as a vehicle for the transmission of these genes locally and potentially across borders via trade. The latter assertion was supported by studies that detected *mcr-1* in five *E. coli* isolates from chicken meat imported to Denmark from another region in Europe [31]. Furthermore, two *mcr-1*-positive *Salmonella* Paratyphi B var. Java isolates were identified in chicken meat imported from mainland Europe to England and Wales [16]. In Tunisia, *mcr-1* was detected in three poultry farms that imported chickens from France or derived their flocks from French chicks [32]. However, it was not clear if the animals were contaminated after their arrival in Tunisia or during subsequent rearing stages. Interestingly, bacteria carrying *mcr* can also contaminate and be transported by other food types such as produce. For example, a study documented the detection of *mcr-1*-positive *E. coli* in vegetables imported to Switzerland from Thailand and Vietnam [33]. As with travelers, the detection of *mcr* in traded food is difficult. Specifically, while imported food is normally screened for microbiological quality and safety, transmissible antibiotic-resistance genes that could be carried by bacteria on food are not typically assessed. Additionally, *mcr* can be carried by nonpathogenic or non-indicator bacteria on otherwise safe food, further complicating their routine detection.

## 5. Conclusions

The World Health Organization (WHO) dubbed antimicrobial resistance a global crisis [34]. Furthermore, predictive models estimated that, by 2050, 300 million human lives could be prematurely lost due to antimicrobial resistance if preventive action is not taken [35]. It was also predicted that the world economy could be affected by AMR, with an estimated loss of USD 60 to 100 trillion by 2050, leading to increased poverty and negative impact on human well-being [35]. Accordingly, it was not surprising that the WHO stated, “There is no time to wait. Unless the world acts urgently, antimicrobial resistance will have disastrous impact within a generation” [34]. Therefore, AMR needs to be tackled with urgency and requires the galvanization and unification of stakeholders around a shared vision of One Health to address the problem globally [34]. This unified effort is important because the world is more connected than ever, and AMR can effectively spread between countries with varying AMR-stewardship programs via disparate mechanisms. In that regard, the global transmission of *mcr* accurately exemplifies the AMR crisis and provides important insights that can be beneficial for monitoring and controlling other emerging AMR-resistance genes.

The studies discussed above provide evidence associating travel and the food trade with the dissemination of *mcr* across national borders. Evidently, *mcr* acquisition during travel occurred even during relatively short visits, and was independent of travelers’ age and health status, as young and otherwise healthy travelers were also found to be carriers of *mcr*. Therefore, travel to- and trade with countries where *mcr* may be prevalent because of weak antimicrobial stewardship and infrastructure merit being considered risk factors for the spread of colistin resistance. The risk may be higher under certain scenarios, including those where countries depend heavily on tourism and/ or imports to boost their economy and meet their food demands, respectively. Additionally, events that engage large multinational gatherings of humans might also pose a high risk. For example, *mcr-1* was detected in individuals who performed the pilgrimage (Hajj) to Mecca. Specifically, *mcr-1*-positive *E. coli* and *Klebsiella pneumoniae* isolates were detected in pilgrims after their return to their home countries of Morocco and Algeria [36]. Overly crowded multinational events may represent a fertile niche for the rapid transmission of *mcr* between individuals who subsequently carry these genes back to their countries.

Much remains unknown about the diversity and molecular epidemiology of *mcr*-containing bacteria. Additionally, it is unclear whether potential bacterial-fitness costs, if any, could affect the carriage and persistence of *mcr*-carrying plasmids in the human gut, food chain, and environment. Interestingly, a controlled study suggested that the carriage of colistin-resistant ESBL-producing *E. coli* and *mcr-1* by travelers could be transient [11]. However, this likely depends on *mcr*-gene homolog/variant, plasmid type, and the absence of exposure to a selective agent. This is important because *mcr*-carrying plasmids were shown to be diverse, and may harbor other genetic elements, including virulence genes and other important antibiotic-resistance genes, which may enhance the persistence of these plasmids in a niche. Admittedly, controlling the dissemination of *mcr* via travel and trade is obviously challenging. For example, testing every asymptomatic traveler or imported-food sample for *mcr* is unfeasible, costly, and probably intrusive in certain cases. However, with the rise of carbapenem-resistant *Enterobacteriaceae* that are becoming endemic in certain regions and that can be transported across national borders [37], preserving the effectiveness of colistin against these bacteria is a global priority at least until viable alternatives are discovered. Therefore, global efforts and investments aimed at reducing *mcr* in countries where these genes are widely distributed are beneficial or necessary to control the spread of these audacious hitchhikers via travelers and the international food trade.
